# Supplementary material for: A theory of working memory without consciousness or sustained activity
Source: eLife. 2017 Jul 18;6:e23871. doi: 10.7554/eLife.23871 (PMC5589417; doi:10.7554/eLife.23871)
Supplement: Supplementary file 1. — DOI: http://dx.doi.org/10.7554/eLife.23871.019 [file elife-23871-supp1.docx]

| Training 🡪 Testing |  | 0.1 – 0.3s | | 0.3 – 0.6s | | 0.6 – 1.55s | | 1.55 – 2.5s | |
| --- | --- | --- | --- | --- | --- | --- | --- | --- | --- |
|  |  | AUC (SEM) | *p* | AUC (SEM) | *p* | AUC (SEM) | *p* | AUC (SEM) | *p* |
| P 🡪 P | Diagonal | 0.53 (0.01) | **.004**^a^ | 0.58 (0.01) | **.001**^a^ | 0.56 (0.01) | **.001**^a^ | 0.52 (0.01) | .058^a^ |
|  | P3b | 0.51 (0.01) | .152^b^ | 0.55 (0.01) | .**003**^b^ | 0.52 (0.01) | .064^b^ | 0.51 (0.005) | .101^b^ |
|  | Maintenance | 0.50 (0.004) | .507^b^ | 0.50 (0.005) | .382^b^ | 0.52 (0.01) | **.046^b^** | 0.51 (0.01) | .382^b^ |
| P 🡪 WM | Diagonal | 0.52 (0.005) | **.003**^a^ | 0.55 (0.01) | **.001**^a^ | 0.53 (0.005) | **.001**^a^ | 0.50 (0.01) | .486^a^ |
|  | P3b | 0.50 (0.004) | .279^b^ | 0.53 (0.01) | **.011**^b^ | 0.51 (0.01) | .101 ^b^ | 0.49 (0.01) | .101^b^ |
|  | Maintenance | 0.49 (0.005) | .**039**^b^ | 0.49 (0.01) | .311^b^ | 0.51 (0.006) | .279 ^b^ | 0.50 (0.01) | .972^b^ |
| WM 🡪 WM | Diagonal | 0.52 (0.01) | .066^a^ | 0.57 (0.02) | **.007**^a^ | 0.55 (0.01) | **.001**^a^ | 0.52 (0.01) | .173^a^ |
|  | P3b | 0.50 (0.01) | .807^b^ | 0.54 (0.01) | **.020**^b^ | 0.50 (0.01) | .807^b^ | 0.49 (0.01) | .422^b^ |
|  | Maintenance | 0.50 (0.01) | .507^b^ | 0.49 (0.01) | .552 ^b^ | 0.51 (0.01) | .650^b^ | 0.51 (0.01) | .600^b^ |
| WM 🡪 P | Diagonal | 0.52 (0.005) | **.010**^a^ | 0.55 (0.01) | **.001**^a^ | 0.53 (0.01) | **.014**^a^ | 0.51 (0.01) | .276^a^ |
|  | P3b | 0.50 (0.006) | .753^b^ | 0.53 (0.01) | **.016**^b^ | 0.50 (0.01) | .972^b^ | 0.49 (0.01) | .463^b^ |
|  | Maintenance | 0.49 (0.01) | .279^b^ | 0.49 (0.01) | .101 ^b^ | 0.51 (0.01) | .701^b^ | 0.50 (0.01) | .972^b^ |

**Table 1. Statistics for decoding analyses**

Statistics are shown for decoding of visibility category (seen vs. unseen) as a function of task and testing time bin. The first column identifies the respective training and testing sets (P = perception task; WM = working memory task), the second column the training classifiers (Diagonal = diagonal, P3b = 300 – 600ms, Maintenance = 0.8 – 2.5s), that were averaged. Bold numbers indicate above-chance decoding performance (^a^one-tailed, ^b^two-tailed Wilcoxon signed-rank test across subjects). AUC = area under the curve; SEM = standard error of the mean (across participants).
