# Supplementary material for: A theory of working memory without consciousness or sustained activity
Source: eLife. 2017 Jul 18;6:e23871. doi: 10.7554/eLife.23871 (PMC5589417; doi:10.7554/eLife.23871)
Supplement: Supplementary file 3. — DOI: http://dx.doi.org/10.7554/eLife.23871.021 [file elife-23871-supp3.docx]

| BF |  | 0.1 – 0.3s: Target | 0.3 – 0.6s: Target | 0.6 – 1.55s: Target | 1.55 – 2.5s: Target | -0.5 – 0: Resp | 0 – 0.8: Resp |
| --- | --- | --- | --- | --- | --- | --- | --- |
| Distractor |  |  |  |  |  |  |  |
| All | Occ | **46.72** | **14.47** | n/a | n/a | n/a | n/a |
|  |  |  |  |  |  |  |  |
| Target |  |  |  |  |  |  |  |
| All | Occ | **109.60** | **125.33** | 2.29 | 0.77 | n/a | n/a |
| P Seen | L Temp | **4.84** | **10.03** | **3.62** | 0.16 | n/a | n/a |
|  | Occ | 0.68 | **496.64** | 0.96 | 0.21 | n/a | n/a |
|  | R Temp | **6.81** | **5256.15** | **23.35** | 0.13 | n/a | n/a |
| Seen | L Temp | **24.07** | **175.81** | **3.89** | 0.17 | n/a | n/a |
|  | Occ | **56.90** | **45.43** | **3.77** | 0.40 | n/a | n/a |
|  | R Temp | 2.31 | **496.24** | 1.22 | 0.38 | n/a | n/a |
| Unseen | L Temp | 2.58 | 1.33 | 0.11 | 0.28 | n/a | n/a |
|  | Occ | **3.34** | 1.53 | 0.11 | 0.30 | n/a | n/a |
|  | R Temp | 0.23 | 0.48 | 0.14 | 0.47 | n/a | n/a |
| Unseen+ | L Temp | **3.75** | 0.22 | 0.46 | 0.21 | n/a | n/a |
|  | Occ | 0.76 | 0.65 | 0.24 | 0.29 | n/a | n/a |
|  | R Temp | 0.13 | 0.13 | 0.18 | 0.16 | n/a | n/a |
| Unseen- | L Temp | 0.49 | 0.32 | 0.13 | 0.34 | n/a | n/a |
|  | Occ | 0.35 | 0.11 | 0.11 | 0.11 | n/a | n/a |
|  | R Temp | 0.12 | 0.19 | 0.14 | 0.28 | n/a | n/a |
|  |  |  |  |  |  |  |  |
| Response |  |  |  |  |  |  |  |
| Seen | L Temp | **11.35** | **41.95** | **4.16** | 0.27 | 0.23 | **90.13** |
|  | Occ | **7.41** | **56.46** | **4.60** | 0.56 | 0.31 | **152.61** |
|  | R Temp | 1.42 | **330.43** | 0.95 | 0.32 | 0.30 | **606.35** |
|  | Frontal | 1.05 | 1.12 | 2.42 | 0.57 | 0.60 | **127.23** |
| Unseen | L Temp | 1.28 | 0.23 | 1.37 | 1.05 | 0.52 | **137.36** |
|  | Occ | 0.44 | 0.25 | 0.98 | 1.32 | 1.24 | **49.11** |
|  | R Temp | 0.18 | 0.22 | 0.23 | 0.35 | 0.35 | **13.73** |
|  | Frontal | 0.35 | **12.69** | 0.47 | **15.86** | 0.32 | **41.58** |
| Unseen+ | L Temp | 0.85 | 0.33 | 2.19 | 0.38 | 2.25 | 2.05 |
|  | Occ | 0.23 | 0.43 | 0.38 | 0.35 | 2.10 | 0.66 |
|  | R Temp | 0.13 | 0.17 | 0.18 | 0.17 | 0.78 | 1.90 |
|  | Frontal | 0.32 | 0.82 | 0.14 | 0.77 | 0.59 | **16.76** |
| Unseen- | L Temp | 1.47 | 0.32 | 1.15 | 0.50 | 0.20 | **5.13** |
|  | Occ | 1.23 | 0.94 | 0.84 | 0.44 | 0.47 | **3.45** |
|  | R Temp | 0.19 | 0.12 | 0.16 | 0.21 | 0.36 | 0.88 |
|  | Frontal | 0.15 | 0.18 | 0.69 | 0.23 | 0.13 | **5.14** |

**Table 3. Bayes Factors for circular-linear correlation analyses**

Bayes Factors for circular-linear correlation analyses between the average amplitude of the MEG signal in the gradiometers and distractor, target, and response position are shown as a function of visibility, accuracy, channel group and time window. The first four time windows are relative to target onset, the last two relative to the onset of the response screen. Bold numbers indicate strong evidence in favor of the alternative hypothesis (i.e., an increase in correlation values relative to an empirical baseline as assessed by a one-tailed Bayesian *t*-test). Frontal = frontal gradiometers; L Temp = left temporo-occipital gradiometers; Occ = occipital gradiometers; P = perception task; Resp = response; R Temp = right temporo-occipital gradiometers; SEM = standard error of the mean (across subjects); Unseen+ = unseen correct trials (within +/- 2 positions of actual target location); Unseen- = unseen incorrect trials.
