# Supplementary material for: A theory of working memory without consciousness or sustained activity
Source: eLife. 2017 Jul 18;6:e23871. doi: 10.7554/eLife.23871 (PMC5589417; doi:10.7554/eLife.23871)
Supplement: Supplementary file 4. — DOI: http://dx.doi.org/10.7554/eLife.23871.022 [file elife-23871-supp4.docx]

| Task | Target | Visibility 4: clearly seen  *M (SD)* | Visibility 3: weakly seen  *M (SD)* | Visibility 2: glimpse  *M (SD)* | Visibility 1: unseen  *M (SD)* |
| --- | --- | --- | --- | --- | --- |
| Perception | Present | 9.8 (16.6) | 17.3 (12.8) | 46 (17.8) | 61.2 (15.4) |
|  | Absent | 0.1 (0.3) | 0.5 (1.4) | 3.0 (3.8) | 28.9 (5.7) |
|  |  |  |  |  |  |
| Working memory | Present | 10.1 (18.0) | 15.8 (14.6) | 45.2 (17.8) | 57.4 (17.4) |
|  | Absent | 0.0 (0.0) | 0.2 (0.6) | 1.9 (1.9) | 29.3 (4.4) |

**Table 4. Trial counts**

Number of trials included in the MEG analyses are listed as a function of task (perception vs. working memory task), target presence (present vs. absent), and visibility rating. Mean (*M*) and standard deviation (*SD*) are based on 13 participants and all trials retained after preprocessing of the MEG data.
